# Nuclear receptor 5A2 regulation of *Agrp* underlies olanzapine-induced hyperphagia

**DOI:** 10.1038/s41380-023-01981-9

**Published:** 2023-02-10

**Authors:** Rizaldy C. Zapata, Dinghong Zhang, Avraham Libster, Alessandra Porcu, Patricia Montilla-Perez, Aisha Nur, Baijie Xu, Zhi Zhang, Stephanie M. Correa, Chen Liu, Francesca Telese, Olivia Osborn

**Affiliations:** 1 Division of Endocrinology and Metabolism, Department of Medicine, University of California San Diego, La Jolla, CA 92093 USA; 2 Department of Psychiatry, University of California San Diego, La Jolla, CA 92093 USA; 3 Department of Drug Discovery and Biomedical Sciences, University of South Carolina, Columbia, SC 29208 USA; 4 Center for Hypothalamic Research, Departments of Internal Medicine and Neuroscience, Peter O’Donnell Jr. Brain Institute, The University of Texas Southwestern Medical Center, Dallas, TX 75390 USA; 5 Department of Integrative Biology and Physiology, University of California Los Angeles, Los Angeles, CA 90095 USA

**Keywords:** Neuroscience, Physiology, Schizophrenia

## Abstract

Antipsychotic (AP) drugs are efficacious treatments for various psychiatric disorders, but excessive weight gain and subsequent development of metabolic disease remain serious side effects of their use. Increased food intake leads to AP-induced weight gain, but the underlying molecular mechanisms remain unknown. In previous studies, we identified the neuropeptide *Agrp* and the transcription factor nuclear receptor subfamily 5 group A member 2 (*Nr5a2*) as significantly upregulated genes in the hypothalamus following AP-induced hyperphagia. While *Agrp* is expressed specifically in the arcuate nucleus of the hypothalamus and plays a critical role in appetite stimulation, *Nr5a2* is expressed in both the CNS and periphery, but its role in food intake behaviors remains unknown. In this study, we investigated the role of hypothalamic *Nr5a2* in AP-induced hyperphagia and weight gain. In hypothalamic cell lines, olanzapine treatment resulted in a dose-dependent increase in gene expression of *Nr5a2* and *Agrp*. In mice, the pharmacological inhibition of NR5A2 decreased olanzapine-induced hyperphagia and weight gain, while the knockdown of *Nr5a2* in the arcuate nucleus partially reversed olanzapine-induced hyperphagia. Chromatin-immunoprecipitation studies showed for the first time that NR5A2 directly binds to the *Agrp* promoter region. Lastly, the analysis of single-cell RNA seq data confirms that *Nr5a2* and *Agrp* are co-expressed in a subset of neurons in the arcuate nucleus. In summary, we identify *Nr5a2* as a key mechanistic driver of AP-induced food intake. These findings can inform future clinical development of APs that do not activate hyperphagia and weight gain.

## Introduction

Antipsychotic (AP) medications are efficacious treatments for various psychiatric disorders [1–6] but excessive weight gain remains a serious side effect of their use [3, 7, 8]. Approximately 20% of patients treated with a broad range of APs gain clinically significant amounts of weight (>7% of their baseline weight) [9]. Drug safety reviews have shown that the percentage of weight gain varies between individuals and depends on the drug, ranging from ~20–40% for olanzapine (OLZ) and clozapine, and ~10–20% for quetiapine and risperidone [9–16]. APs induce weight gain in human [7, 17–19] and rodents [20–29] by increasing food intake (hyperphagia). However, little is known about the molecular mechanisms by which APs induce hyperphagia. Previous studies have relied on non-specific anti-obesity drugs that suppress basal feeding to reduce AP-induced weight gain (i.e., locaserin [30], orlistat [31], liraglutide [32], nizatidine [33] metformin [34]). While using anti-obesity drugs in combination with APs is clinically beneficial to offset weight gain, they do not shed light on the specific mechanisms underlying AP-induced hyperphagia. Delineating the specific mechanisms driving AP-induced hyperphagia can inform future drug development of highly effective APs without this serious adverse effect and, more broadly, anti-obesity drugs.

In our previous work on AP-induced hyperphagia in *C. elegans* and mice, we identified gene expression programs altered by AP-induced hyperphagia. The agouti-related peptide *Agrp* and the transcription factor nuclear receptor subfamily 5 group A member 2 (*Nr5a2*) genes were significantly upregulated following AP-induced hyperphagia.

The *Agrp-*expressing neurons in the arcuate nucleus (ARC) of the hypothalamus play a major role in food intake behavior [35–39]. While some studies have reported increased expression of *Agrp* after AP-treatment [25, 40, 41], the molecular mechanisms regulating the AP-induced expression of this key pro-feeding gene are not well understood [42–44]. In contrast, *Nr5a2* is broadly expressed throughout the body and has well-described roles in the liver [45, 46], gut [47], and pancreas [48, 49]. *Nr5a2* has also been implicated in adipocyte formation [50], intestinal function [47] pancreatic inflammation [48] and expression of pancreatic digestive enzymes [51, 52]. However, little is known about its role in the brain [53, 54]. Within the brain [55], *Nr5a2* expression is enriched in the ARC of the hypothalamus [53, 56–58] and single-cell analysis has revealed that *Nr5a2* expression marks a specific subset of neurons in this region [59]. Our previous studies provided the first insights into the potential involvement of *Nr5a2* in AP-induced food intake [26]. In these *C.elegans* based studies, we determined that *Nr5a2* ortholog/nhr-25 mutant strain (nhr-25(ku215)) was resistant to AP-induced hyperphagia [26]. In the current study, we used several mouse models to investigate the role of *Nr5a2* in AP-induced food intake and weight gain.

## Materials and methods

### In vitro studies

Adult mouse hypothalamic cell lines (mHypoA-59, CLU468 cells, Cedarlane) were cultured as described previously [60, 61], and confirmed to be *Mycoplasma*-free. In brief, cells were grown and maintained in high-glucose, pyruvate-free DMEM supplemented with 10% fetal bovine serum, L-glutamine (Cat. 25030081, Gibco, NY), and 10 u/ml of penicillin and 10 ug/ml of streptomycin (Cat. 15149-122, Gibco) of in a 5% CO_2_ environment. Cells were treated with OLZ (O0393, TCI Americas) (25–100 µM) for 6 h for mRNA expression analysis of NR5A2 and AGRP. For protein analysis, cells were incubated with 100 µM OLZ for 24 h. For NR5A2 protein nuclear expression studies, cells were co-treated with an NR5A2 antagonist SR1848 (AOB1355, Aobious, Gloucester, MA) for 24 h at 5 µM in DMSO as described previously [62]. For *Agrp* mRNA expression, cells were incubated with 1–5 µM SR1848 for 6 h, while for AGRP protein expression, cells were incubated with 1 µM of SR1848 for 24 h.

### Gene expression

RNA isolation was performed using Trizol (cat # 15596026, Thermo Fisher) and was purified using RNeasy Plus Mini Kit (cat # 774104, Qiagen) using the manufacturer’s recommendations. cDNA was reverse transcribed from 300 ng of RNA using High-Capacity cDNA transcription kit (cat # 4368813, Applied Biosystems). Relative expression was analyzed by qPCR using StepOne Realtime PCR System. Gene expression was calculated after normalization to the housekeeping genes [63] (Pgk1, Hprt1) using the Δ^ΔCt^ method. Gene expression was calculated relative to experimental controls. Primer sequences (5’-3’) used to measure gene expression are listed in Table 1.

**Table 1:** Primer sequences.

Gene	Primer sequence 5’-3’
*Agrp*	F: GGAACAGTGTTTTCTGCTCCC R: ACTCGTGCAGCCTTACACAG
*Npy*	F: TAACAAGCGAATGGGGCTGT R: TTCAAGCCTTGTTCTGGGGG
*Pomc*	F: GGCGACGGAAGAGAAAAGAGG R: TGTTCAGTCTCCTGCCTGTCG
*Cart*	F: TGGATGATGCGTCCCATG R: TACTTCTTCTCATAGATCGGAAT
*Nr5a2*	F: AGTCTGAGGTTTCCTTCCCAAAG R: CTAGAGCAAGCTTCCAGGGG
*Pgk1*	F: CTGACTTTGGACAAGCTGGACG R: GCAGCCTTGATCCTTTGGTTG
*Hrpt1*	F: CACAGGACTAGAACACCTGC R: GCTGGTGAAAAGGACCTCT
*Prox1*	F: CAGCGGACTCTCTAGCACAG R:GCCTGCCAAAAGGGGAAAGA
*Satb2*	F: AAGGCCGTGGGAGGTTTGAT R: GCACATCTTTCCGCACCAAG
*Sox4*	F:CACAACGCCGAGATCTCCAA R:CCCGACTTCACCTTCTTTCG
*Ctip2*	F:AAGCTGGGGTTCTCTCTTGC R: ATTGAGGCAGGCCACGTAAA
*Jak3*	F: GCCCCACCGAGGTTCAG R: GAGAGGAAGCTGCGGGTCTA
*Stat1*	F: GATCGCTTGCCCAACTCTTG R: ACTGTGACATCCTTGGGCTG
*Nmi*	F:GCCAGGTTAGTGTTTTCGAGG R: CTACAGAACTCAGCACCCGC
*Cyp8b1*	F:TTGCAAATGCTGCCTCAACC R: TAACAGTCGCACACATGGCT

### Western blots

Hypothalamic proteins were isolated using NP-40 with 0.03 M PMSF and cOmplete™, EDTA-free Protease Inhibitor, (Roche 11873580001). Nuclear proteins were isolated from ~6 million HypoA cells treated with either vehicle or 5uM SR1848 for 24 h [62]. Briefly, cells were incubated with a hypotonic solution (10 mM HEPES pH 7.9, 1.5 mM MgCl_2_, 10 mM KCl, 1 mM DTT, protease inhibitors) for 20 min to isolate cytoplasmic proteins followed by a high salt buffer (20 mM HEPES pH 7.9, 420 mM NaCl, 1.5 mM MgCl_2_, 1 mM DTT, 25% glycerol, 0.5% Igepal, protease inhibitors) for 50 min to extract nuclear proteins. Proteins were then fractionated in 4–15% Mini PROTEAN TGX acrylamide gels, transferred to PVDF, blocked with 5% BSA, incubated with the primary antibody overnight and secondary antibody for 60 min before detection using ECL (SuperSignal, Thermo Fisher 34580, 34095). Band intensities were quantified using densitometry in ImageLab (Biorad). The following antibodies were used to detect proteins: anti-AGRP (1:100, sc-518077, Santa Cruz Biotechnology), anti-NR5A2 (1:1000, PP-H2325-00, RnD Systems), anti-caveolin (1:1000, 610407, BD Transduction Lab), anti-beta actin (1:2000, 3700, Cell Signaling), anti-histone 3 (1:1000, 4499, Cell Signaling), anti-mouse IgG (1:4000, 115035003, Jackson Immunoresearch), anti-rabbit IgG (1:4000, NA934V, GE Healthcare).

### Mice

All protocols were approved by UCSD IACUC. All mice were singly housed in standard cages and acclimated to laboratory conditions (12:12 light–dark, 20–21 °C, 50% humidity) for 7 days before experimentations. Mice were singly housed to accurately measure daily food intake by weighing food in the hopper and accounting for any spillage [64]. All studies were performed in female C57B6/J mice (Jackson, stock # 000664) or *Agrp* null mice. *Agrp*^−/−^ (KO) mice were gifted by Dr. Chen Liu of UT Southwestern. Sample sizes were based on previous similar studies [28, 29, 65, 66]. Mice were randomized and all groups were weight-matched prior to any drug treatment or surgeries. Furthermore, the metabolic phenotyping was conducted by one postdoctoral fellow, and no blinding was incorporated.

### Olanzapine administration in diet

OLZ was compounded into 45% HFD diet (54 mg/kg = ~6–8 mg/kg) as a convenient dosing strategy [26, 28, 29], and this approach has been used in many other studies investigating AP-induced hyperphagia and weight gain [25, 65, 67–69] where HFD feeding potentiates OLZ-induced hyperphagia and weight gain. This dose results in mouse plasma levels (21 ± 5 ng/ml) that are similar to levels observed in humans treated with OLZ (10–50 ng/ml) [65].

### Systemic inhibition of NR5A2

Twelve-week old female mice were acclimated to receive intraperitoneal (IP) injections of sterile saline for 3 days before the experiment and then were randomized to receive a 45% high-fat diet (CON, D09092903B, Research Diets) with or without OLZ (54 mg/kg, D16111030). Mice were then further randomized to receive the vehicle solution (VEH, 10% DMSO, 10% Tween 80 in 0.9% NaCl) or the NR5A2 inhibitor (SR1848) at 30 mg/kg daily for 7 days. Food intake and body weight were measured daily. Animals were sacrificed at the end of the study and the hypothalamus was dissected, snap-frozen in liquid nitrogen, and stored at −80 °C until analyses.

### Pair feeding studies

Female mice 10–12 weeks of age were divided into three groups: (1) OLZ diet, ad libitum + vehicle, (2) OLZ diet, ad libitum + SR1848 (30 mg/kg), (3) OLZ diet and fed the same average quantity of food that group 2 ate (pair fed) + vehicle. All mice consumed the OLZ diet (54/mg/kg) for the 7 day study.

### Hypothalamic inhibition of *Nr5a2*

Twelve-week old female mice were anesthetized with 5% isoflurane and placed in a stereotactic apparatus (David Kopf Instruments, Model 900HD Motorized Small Animal Stereotaxic). Brain injections were performed in mice under a continuous flow of 2% isoflurane. Correct targeting of the arcuate nucleus of the hypothalamus was confirmed by injection of AAV2-eSyn-EGFP-wpre (Vector Biolabs) using co-ordinates (A–P: −1.58 mm from Bregma; M–L ± 0.25 mm from midline; D–V: −5.8 mm into the skull). *Nr5a2* siRNA (SMARTpool: Catalog ID: L-047044-01-0005, Dharmacon, Lafayette, CO) or non-targeting control siRNA (Catalog ID:D-001810-10-05; Dharmacon) (*n* = 4–5) was delivered bilaterally (200 nl of siRNA) into the ARC using coordinates above. To allow time for diffusion, the injection needle remained immobile for 10 min before removal. Mice were allowed to recover for 7 days before transitioning to CON or OLZ treatment. Food intake was measured daily and body weight every other day for 14 days, at which point many studies have established significant OLZ-induced hyperphagia and weight gain [26, 28, 29, 66, 70]. Animals were sacrificed at the end of the study and the hypothalamus was dissected, snap-frozen in liquid nitrogen, and stored at −80 °C until analyses.

### *Agrp* null studies

Twelve-week old WT and KO female mice were randomized to receive either CON or OLZ (*n* = 9–17/group). Food intake was measured daily, and body weight was every other day for 12 days. Animals were sacrificed at the end of the study and the hypothalamus was dissected, snap-frozen in liquid nitrogen, and stored at −80 °C until analyses.

### Photometry experiments

Female *Agrp*-ires-cre mice (Jackson Laboratory stock no. 012899), aged 12–20 weeks, received viral infusions (200–300 nl of AAV1-CAG-Flex-GCaMP6s-WPRE-SV40 (Addgene, #100842) into the arcuate nucleus to express GCaMP6s, a potent genetically encoded calcium indicator [71]. Ceramic optical fiber ferrules (400 µm core diameter, 2.5 mm ferrule diameter, NA 0.39, RWD, R-FOC-L400C-39NA) were inserted just dorsal (~300 µm above) to the injection site and fixed to the skull by applying a thin layer of C&B Metabond (Parkell) onto the skull surface, followed by building a cap using dental cement (Ortho-Jet, Lang Dental). Animals were allowed to recover for 2–3 weeks before recordings. Calcium imaging from *Agrp* neurons was conducted using a commercially available system (FP3002, Neurophotometrics) according to the manufacturer’s instructions. For fiber photometry using GCaMP6s in *Agrp* neurons, successful targeting of *Agrp* neurons was tested 2–3 weeks after surgery by fasting the mice for 6–24 h and then refeeding with chow and observing a significant drop in *Agrp* activity. To measure *Agrp* responsiveness to OLZ or Veh, mice were injected IP with OLZ (0.5 mg/kg/200 ul volume) or saline based on previous studies [72]. Video of the recording session was collected using a webcam, and was synchronized to the recorded photometry signal. All the recorded traces were aligned relative to the time the mouse was returned to the home cage. Analysis of photometry data was based on previously published methods [73].

### Chromatin immunoprecipitation (ChIP)

ChIP experiments were conducted in triplicates using methods previously described in other neuronal cell types [74–76]. Briefly, hypothalamic mHypoA-59 cells were grown in 10 cm dishes and at 75–80% confluency and fixed with 1% formaldehyde. Nuclei were isolated before chromatin extraction. Chromatin from ~10 million cells was sheared using a sonication device (Bioruptor Pico, #2013–2019, Diagenode) and optimized to produce ~400 bp fragments. Chromatin was immunoprecipitated using 4 ug of NR5A2 antibody (PP-H2325-00. 5 μg/ChIP, RD Biosystems) [48] and 20 ul of beads without any antibody were used as control samples. Importantly, this antibody has been validated in the *Nr5a2* KO [77] and has successfully been used in liver [78, 79] and pancreatic [48] ChIP experiments in mice. After primary and secondary antibody incubation and washes, purified DNA was used in quantitative PCR reactions with primers targeting the promoter of *Nr5a2* target gene Prospero Homeobox 1(Prox1) promoter (Prox1-F 5’-CTGTTAACTGTGCCCAGGGAGAGGA-3’, Prox1-R 5’-TGGTTTGACATCTTGGGTGA-3’) [54] as a positive control [54] Prox1_LocA-F: 5’-GTATCTTCACCCGGTTGCTG-3’ Prox-1-LocA-R: 5’-CGATTCATGTAAATAACACC-3’ as a negative control [54], or the *Agrp* promoter region (*Agrp*-F 5’-GGGGTCTGGACACCCTATCT-3’, *Agrp*-R 5’-CACACGTGACTGCTTCCTGT-3’) [80]. Fold enrichment was calculated relative to the no-antibody control samples. Notably, previous published immunohistochemistry and western blot studies have established that Prox 1 is expressed in the rodent hypothalamus [81].

### RNAscope

WT female C57B6/J mice were anesthetized with Pentobarbital and then transcardially perfused with 20 ml PBS followed by 20 ml 4% PFA (Sigma). Brains were removed and incubated in 4% PFA overnight at 4 °C. Following cryoprotection in 30% sucrose, 25 μm coronal sections of the brains were collected using a sliding microtome (Leica SM2010R) and stored at −20 °C in a cryoprotectant solution. On the day of RNAScope staining, slices containing the arcuate nucleus were selected for staining and washed 3 times in PBS and then mounted on glass slides (VWR). RNAscope was performed using the RNAscope Multiplex Fluorescent Reagent Kit v2 (ACD, #323100) following the user manual (ACD, USM-323100) with minor modifications. Tissue sections corresponding to ARC from 3 VEH and 3 OLZ mice (1 section per mouse) were hybridized with a mix of two probes; Agrp (ACD, # 400711-C2)  + Fos (ACD, # 316921). We used DAPI as a nuclear stain. To assess both tissue RNA integrity and assay procedure, a separate group of sections was incubated with negative probes (data not shown). Images were acquired with the Keyence fluorescence microscope and analyzed with the ImageJ software. The number of Agrp/fos positive cells was manually counted from six sections per mouse, *n* = 6.

### Cell type-specific expression analysis of *Nr5a2* and *Agrp* using single-cell RNA-seq data

The dotplot visualization of *Nr5a2* and *Agrp* expression across 34 neuronal sub-cluster of the ARC was produced using the “explore” function on the Broad Institute single cell portal [82] using Campbell et al. [59] study.

## Results

### Olanzapine treatment increases the expression of *Nr5a2* and *Agrp*

**Fig. 1:**
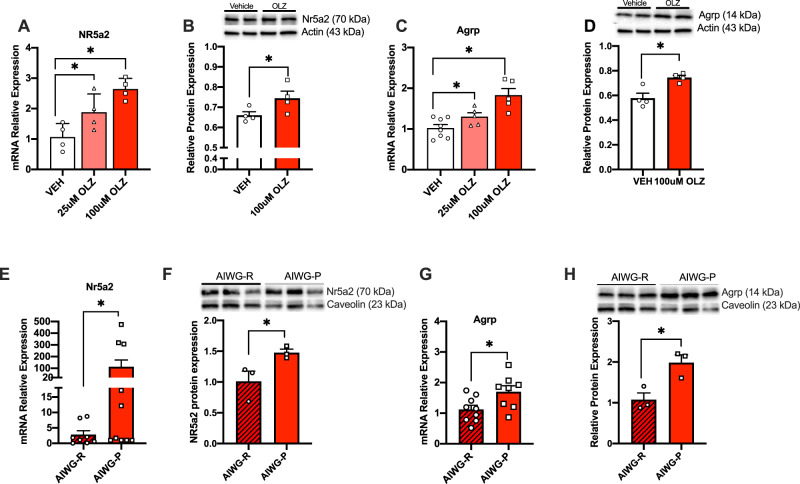
OLZ treatment is associated with elevated *Nr5a2* expression. OLZ treatment of hypothalamic cells results in dose-dependent increase in expression of *Nr5a2* (**A**, **B**) and *Agrp* (**C**, **D**) as determined by qPCR (**A**, **C**) and western blotting (**B**, **D**). Mice that are highly *Prone* to Antipsychotic-Induced Weight Gain (AIWG-P) have significantly elevated hypothalamic levels of *Nr5a2* (**E**, **F**) and *Agrp* (**G**, **H**) gene expression compared with AIWG-*Resistant* mice (AIWG-R) at both the gene expression (**E**, **G**) and protein level (**F**, **H**). **E** Data passed the Shapiro–Wilk test for normality and is expressed as mean ± SEM and was analyzed using either one-way ANOVA followed by two-stage linear step-up procedure of Benjamini, Krieger and Yekutieli with a false discovery rate of 0.10 (**A**, **C**, *n* = 3–8 replicates per group) or Student’s *t* test (**B**, **D**–**H**, *n* = 3–10 replicates per group), * denotes statistical significance at *p* < 0.05.

To study the effects of AP on *Nr5a2* and *Agrp* expression, we used OLZ treatment in cell lines and mice. While OLZ is associated with a very high risk for weight gain, it is also regarded as one of the most clinically effective medications [83]. OLZ treatment of hypothalamic cells resulted in a significant dose-dependent upregulation of transcript and protein levels of *Nr5a2* (Fig. 1A, B) and *Agrp* (Fig. 1C, D) compared to vehicle treatment. To assess the effect of OLZ in mice, we measured the expression of *Nr5a2* and *Agrp* in the hypothalamus of mice that were defined as prone (gained 6.3 g body weight) or resistant (gained 1.3 g body weight) to antipsychotic-induced weight gain (AIWG) following OLZ treatment [28]. In addition to the previously noted elevation in the hypothalamic expression of *Agrp*, we also observed a highly significant elevation of *Nr5a2* (Fig. 1E, F) and *Agrp* (Fig. 1G, H) gene and protein expression in the AIWG-prone compared with the AIWG-resistant mice. In addition, AIWG-prone mice were also hyperphagic compared to AIWG-resistant mice. Thus, the elevated hypothalamic expression of *Nr5a2* and *Agrp* in the AIWG-prone mice further suggests that these genes may play a role in AP-induced hyperphagia and weight gain.

### NR5A2 inhibitor treatment reduces OLZ-induced food intake and weight gain

**Fig. 2:**
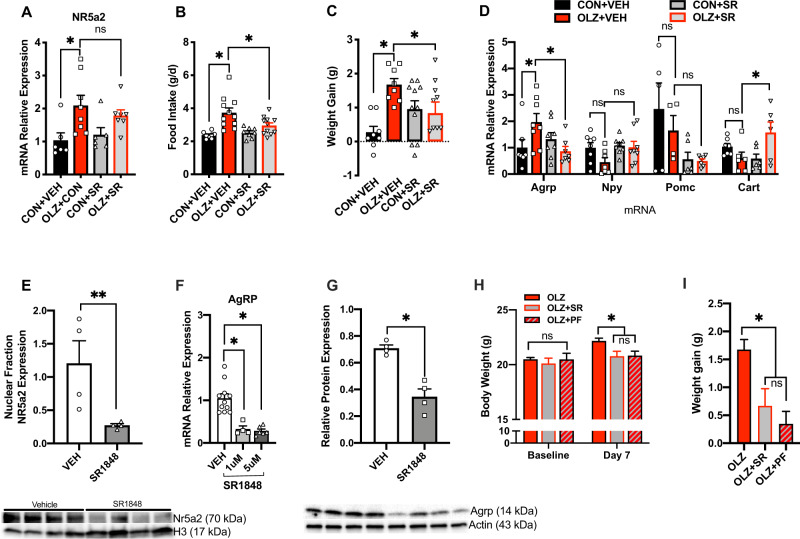
Systemic NR5A2 antagonist treatment reduces food intake and weight gain in mice treated with olanzapine. **A** *Nr5a2* expression, **B** average daily food intake, **C** weight gain, **D** hypothalamic neuropeptide expression in C57BL/6 WT female mice fed either control diet (CON) or OLZ diet and injected with NR5A2 antagonist (SR1848, “SR”, 30 mg/kg) or vehicle (VEH) for 7 days determined by real time RT-qPCR assays. **E** NR5A2 expression in nuclear fraction of hypothalamic cells lines treated with SR1848 (5 μM for 24 h). **F** Quantitative PCR determination of *Agrp* expression in hypothalamic cells treated with SR1848 (1 or 5 μM) for 6 h. **G** AGRP protein expression in hypothalamic cells treated with SR1848 (1 μM for 24 h). Data passed the Shapiro–Wilk test for normality and are expressed as mean ± SEM and was analyzed using either one-way ANOVA followed by two-stage linear step-up procedure of Benjamini, Krieger and Yekutieli with a false discovery rate of 0.10 (**A**–**D**, **H**, **I**, *n* = 7–10), or Student’s *t* test (**E**–**G**, *n* = 4–11 replicates per group), * denotes statistical significance at *p* < 0.05. ** denotes statistical significance at *p* < 0.01.

We further investigated the role of *Nr5a2* in OLZ-induced hyperphagia in mice using a specific NR5A2 antagonist (SR1848, IP 30 mg/kg daily) [62]. SR1848 inhibits NR5A2 function by triggering translocation of NR5A2 from the nucleus to the cytoplasm, which ultimately abrogates its ability to transduce transcription of its targets [62]. While OLZ treatment resulted in elevated hypothalamic expression of *Nr5a2*, co-treatment with SR1848 (OLZ + SR) did not impact *Nr5a2* expression levels (Fig. 2A). In contrast, co-treatment of OLZ with SR1848 resulted in significantly reduced daily food intake (Fig. 2B) and weight gain (Fig. 2C) compared with OLZ alone over 7 days of treatment. Furthermore, hypothalamic levels of *Agrp* (Fig. 2D) were significantly reduced by co-treatment, while other appetite regulating neuropeptides *Npy and Pomc* levels were not significantly changed. As previously shown, SR1848 triggers cytoplasmic translocation of *Nr5a2* from the nucleus in epithelial cells [62]. We have replicated this experiment in hypothalamic cells and show that SR1848 treatment results in significantly lower levels of nuclear *Nr5a2* protein compared with vehicle treatment (Fig. 2E). To determine whether SR1848 has a direct effect on hypothalamic gene expression, we treated hypothalamic cell lines with SR1848 and measured *Agrp* gene expression (Fig. 2F). We observed a significant reduction in *Agrp* expression levels after SR1848 dosing suggesting inhibition of *Nr5a2* in the hypothalamus impacts *Agrp* gene expression. Furthermore, protein levels of *Agrp* (Fig. 2G) were also significantly decreased after SR1848 treatment in hypothalamic cells.

To further support that SR1848 modifies gene expression in the hypothalamus, we treated mice with SR1848 and then extracted the hypothalamus and measured *Nr5a2* target gene expressions. *Nr5a2* target genes were selected from studies comparing the gene expression profiles of embryonic (E12.5) WT and *Nr5a2*KO mice [54] or similar in vitro studies with SR1848 [62]. We selected a subgroup of *Nr5a2* target genes also expressed in the hypothalamus, according to the Allen brain atlas, which included *Prox1, Satb2, Sox4, Ctip2, Jak3, Stat2 Nmi*, and *Cyp8b1*. We found that SR1848 treated mice have decreased expression of *Prox1* (a known direct target of *Nr5a2*) as well as lower expression levels of *Satb2, Sox4, Ctip2 and Cyp8b1* and increased expression of *Jak3, Stat2* and *Nmi* (Supplementary Fig. 1A) in line with the previous KO and SR1848 treatment studies [62]. Therefore, the peripheral administration of SR1848 resulted in specific changes in hypothalamic *Nr5a2* target genes, further suggesting SR1848 impacts hypothalamic gene expression. Notably, because of the location of the ARC, it is conceivable that SR1848 could impact cells in this area without crossing the blood-brain barrier. However, SR1848 is likely to penetrate the blood-brain barrier based on predictions using a computational software [84].

In a previous study, we did not find any significant effects of OLZ on energy expenditure during the dark and light phases using indirect calorimetry [26]. In addition, SR1848 had no effect on the gene expression of thermogenic genes in the brown adipose tissues (Supplementary Fig. 1B). To determine the contribution of SR1848-induced changes in food intake on body weight gain, we also conducted a pair feeding (PF) study. PF mice were then given the same amount of food that the OLZ + SR1848 treated mice consumed. After a week of treatment, OLZ + PF gained less weight than OLZ + VEH (Fig. 2H), resulting in similar blunting of body weight gain in both OLZ + SR and OLZ + PF groups (Fig. 2I). These PF studies suggest that food intake is the dominant physiological mechanism resulting in a reduction of weight gain in the SR1848-treated mice.

#### The knockdown of *Nr5a2* in the arcuate nucleus partially reversed olanzapine-induced hyperphagia and weight gain

**Fig. 3:**
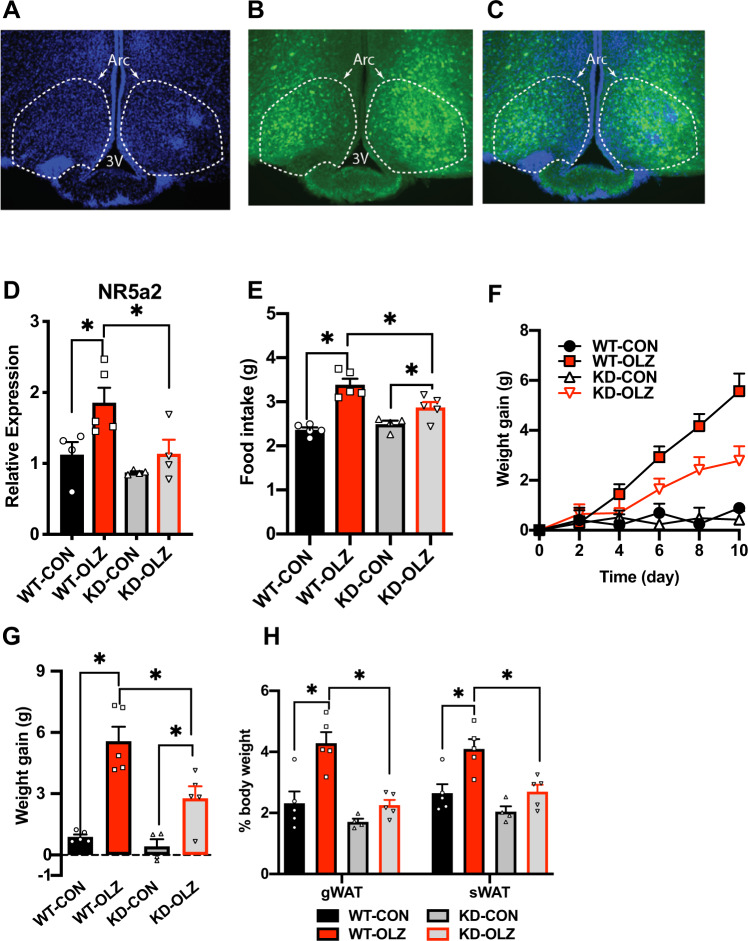
Hypothalamic knockdown of *Nr5a2* significantly bunts OLZ-induced food intake and weight gain. **A**–**C** Bilateral injection of AAV2-eSyn-EGFP (120–150 nl) into the ARC (A–P: −1.58 mm from Bregma; M–L ± 0.25 mm from midline; D–V: −5.8 mm into the skull) (Blue: DAPI, Green: GFP, white dotted lines show ARC boundaries). siRNA-mediated knock down of *Nr5a2*, delivered by stereotaxic injection to the arcuate nucleus, results in **D** reduced expression of *Nr5a2*, **E** blunted OLZ-induced food intake and **F**, **G** reduced OLZ-induced weight gain and body fat (**H**), Data passed the Shapiro–Wilk test for normality and are expressed as mean ± SEM and were analyzed using either one-way ANOVA followed by two-stage linear step-up procedure of Benjamini, Krieger and Yekutieli with a false discovery rate of 0.10 (**D**–**H**, *n* = 4–5), * denotes statistical significance at *p* < 0.05.

To determine whether OLZ-induced food intake and body weight regulation require the expression of *Nr5a2* specifically in the hypothalamus, we used siRNA-mediated knockdown of *Nr5a2* expression in the hypothalamus. siRNAs were delivered directly to the hypothalamus by stereotaxic injection (Fig. 3A–C). As expected, *Nr5a2* expression was increased by OLZ treatment, and treatment with siRNA targeting *Nr5a2* significantly reduced *Nr5a2* expression (Fig. 3D). OLZ treatment increased food intake (Fig. 3E) and body weight gain (Fig. 3F, G) which was reversed by hypothalamic *Nr5a2* siRNA treatment. Furthermore, gonadal (gWAT) and subcutaneous (sWAT) fat mass were also significantly elevated by OLZ treatment and significantly reduced by *Nr5a2* siRNA compared to siRNA control (Fig. 3H).

### Genetic deletion of *Agrp* in mice prevented olanzapine-induced hyperphagia and weight gain

**Fig. 4:**
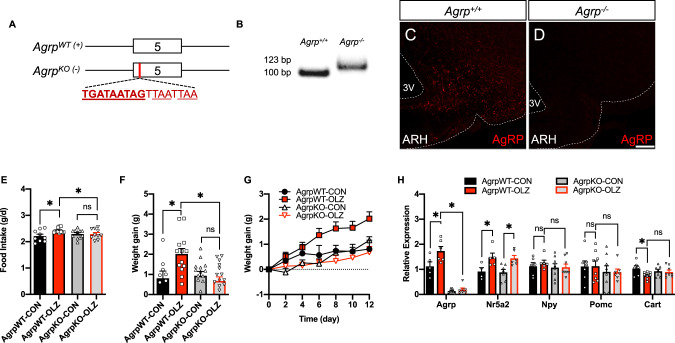
*Agrp* KO mice are resistant to OLZ-induced hyperphagia and weight gain. Generation and characterization of *Agrp*^*−/−*^ mice, **A** Schematic of the wildtype (WT, +) and knockout (KO, −) alleles. Multiple in-frame (underlined bold) and out-of-frame (underlined) stop codons are inserted into the coding sequences in the exon 5 of *Agrp*^−/−^ mice. **B** PCR genotyping of *Agrp*^+/+^ and *Agrp*^−/−^ mice. **C** Immunostaining of AGRP proteins in the ARC of *Agrp*^+/+^ and **D** *Agrp*^−/−^ mice. 3V the third ventricle, ARC arcuate nucleus of the hypothalamus. Scale bar is 50 µm. **E** Food intake, **F**, **G** weight gain, **H** hypothalamic gene expression in WT and KO mice treated with CON or OLZ diets. Data passed the Shapiro–Wilk test for normality and are expressed as mean ± SEM and were analyzed using either one-way ANOVA followed by two-stage linear step-up procedure of Benjamini, Krieger and Yekutieli with a false discovery rate of 0.10 (**E**–**H**, *n* = 9–12 per group), * denotes statistical significance at *p* < 0.05.

Since OLZ treatment increases the expression of *Agrp* similar to *Nr5a2*, we used *Agrp*^−/−^ mice (Fig. 4A–D) to test whether *Agrp* is necessary for the hyperphagic effect of OLZ. As expected, OLZ treatment of WT mice induced higher food intake (Fig. 4A) and weight gain (Fig. 4B, C) compared with control-treated mice. However, KO mice were resistant to the hyperphagic and weight gain response to OLZ treatment (Fig. 4A–C). While OLZ treatment resulted in elevated hypothalamic transcriptional levels of *Nr5a2* in KO mice compared with control-treated KO mice, the expressions of *Npy*, *Pomc* and *Cart* were unchanged (Fig. 4D), suggesting that *Nr5a2* may be upstream of *Agrp* regulation. We also tested whether *Agrp* neurons increase their activity in response to acute OLZ injection. By expressing a genetically encoded calcium indicator (GCaMP6s) and positioning an optic fiber above the injection site (Supplementary Fig. 2A, B), we recorded their activity in vivo (Supplementary Fig. 2C, D). After the injection of OLZ (IP, 0.5 mg/Kg), we did not observe a difference in the activity of *Agrp*-expressing neurons compared to the control group (Supplementary Fig. 2E). These results suggest that the effects of chronic OLZ treatment on weight gain and its dependence on *Agrp* expression do not depend on acute changes in the activity of *Agrp* neurons. To further support this conclusion, we conducted RNAscope analysis of *Fos* expression in Agrp-positive neurons following acute OLZ treatment. We observed that only 20% of the *Argp* neurons co-expressed Fos without significant changes between vehicle and OLZ-treated groups (Supplementary Fig. 2F, G).

### *Nr5a2* directly binds *Agrp* promoter

**Fig. 5:**
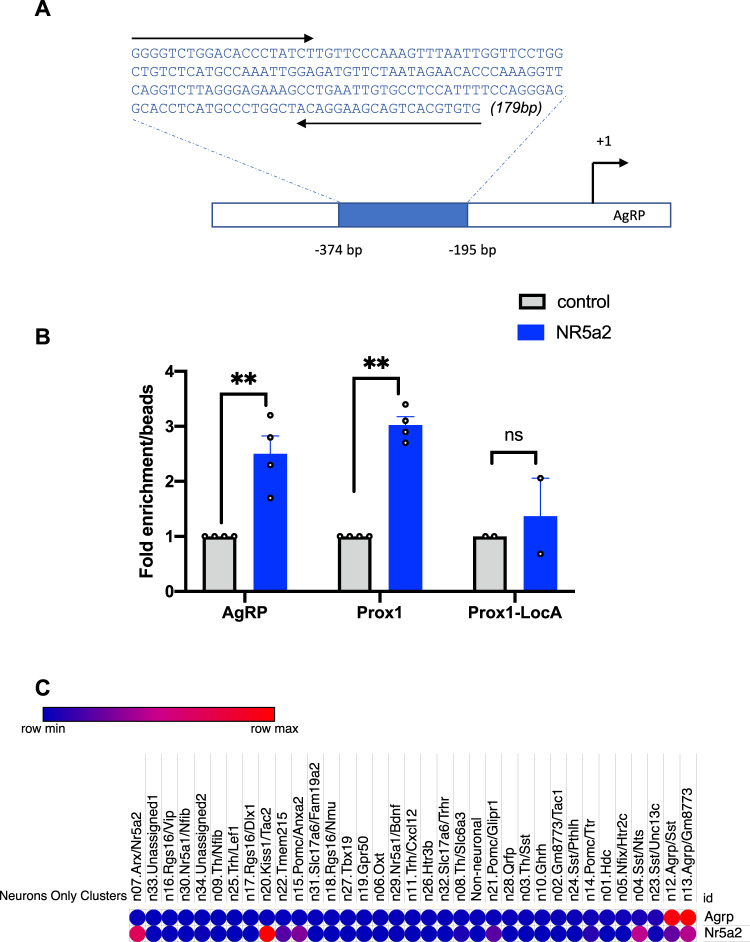
*Agrp* is a direct transcriptional target of *NR5A2*. **A** *Agrp* promoter region. Chromatin immunoprecipitation with NR5A2 antibodies followed by PCR (ChIP-PCR) in the hypothalamic cell line (mHypo-59A) result in enrichment of binding to the **B**. *Prox1* promoter (positive control), Prox1 LOCA (negative control), *Agrp* promoter region compared with beads without antibody. Data are expressed as mean ± SEM and were analyzed using Student’s *t* test (*n* = 2–4 replicates per group), ** denotes statistical significance at *p* < 0.01. **C** Expression of *Agrp* and *Nr5a2* across ARC-ME neuronal populations from GEO dataset GSE93374. Scaling is relative to each gene’s expression across all cells in a given annotation selection, i.e., cells associated with each column label in the dot plot.

These data led us to hypothesize that *Nr5a2* may directly regulate the expression of *Agrp* by binding to its promoter. To test this, we conducted chromatin immunoprecipitation with *Nr5a2* antibodies followed by PCR (ChIP-PCR) in hypothalamic mHypo-A59 cells. In agreement with previous studies in neuronal stem cells [54], we found that *Nr5a2* binds the Prospero Homeobox 1(Prox1) promoter (Fig. 5A). We then used primers specific for *Agrp* promoter region [80] and determined ~2.5-fold enrichment of *Nr5a2* binding to the *Agrp* promoter region over the control sample (Fig. 5B). As a negative internal control, we included a region of the Prox1 promoter where *Nr5a2* does not bind (Prox-LocA) and show no enrichment for *Nr5a2* binding in this region (Fig. 5B). These ChIP experiments in hypothalamic cells identify *Agrp* as a direct transcriptional target of the transcription factor *Nr5a2*. To further explore the relationship between *Nr5a2* and *Agrp*, we analyzed an available single-cell transcriptomic study of the ARC [59] (GSE93374), which identified 34 clusters of molecularly distinct neuronal subtypes. In this dataset, *Nr5a2* expression defines one of these subsets (n07.*Arx/Nr5a2*). We used this dataset to examine the expression of *Agrp* and *Nr5a2* across the ARC sub-neuronal populations (Fig. 5C). This analysis confirmed the previous observations that *Nr5a2* is highly expressed in *Kiss1* neurons, but importantly also revealed that *Nr5a2* is also co-expressed in *Agrp* populations referred to as n13.*Agrp/Gm8773*. Therefore, these studies suggest that a specific population of *Nr5a2*-expressing cells co-express *Agrp* in the ARC, which play a major role in OLZ-induced hyperphagia and weight gain.

## Discussion

In these studies, we used several mouse models to investigate the role of *Nr5a2* in OLZ-induced food intake and weight gain. We first determined that OLZ treatment resulted in a dose-dependent increase in *Nr5a2* and *Agrp* expression in mouse hypothalamic cells. Furthermore, hypothalamic *Nr5a2* expression was highly induced in mice that were particularly prone to AIWG compared with mice that were relatively protected from AIWG. Administration of SR1848, a specific NR5A2 inhibitor, decreased OLZ-induced hyperphagia and weight gain, and the knockdown of *Nr5a2* in the ARC partially reversed OLZ-induced hyperphagia. Importantly, *Agrp* null mice were protected from OLZ-induced hyperphagia and weight gain, despite having elevated hypothalamic *Nr5a2* expression, suggesting this transcription factor may regulate *Agrp* expression. The ChIP-PCR results reported in the current study show, for the first time, that NR5A2 directly binds to the *Agrp* promoter region and suggest that *Nr5a2* directly regulates the expression of this pro-feeding neuropeptide in the hypothalamus. Single-cell RNA-seq studies [59] confirm that *Nr5a2* and *Agrp* are co-expressed in a subset of neurons in the ARC.

Despite the importance of *Agrp* in the homeostatic control of feeding, the transcriptional regulation of its expression is still poorly understood. Studies have shown that *Agrp* transcription is regulated by key energy sensors, including peroxisome proliferator-activated receptor gamma coactivator 1-alpha [85], AMP-activated protein kinase or sirtuin 1, and estrogen receptor alpha and signal transducer and activator of transcription 3 [86], forkhead box protein O1 [87], Krüppel-like factor 4 [88]. Our studies discovered a new transcriptional regulator, NR5A2, to this important list of factors that can regulate *Agrp* expression. Future ChIP-seq studies are warranted to determine the comprehensive transcriptional targets of NR5A2 in *Agrp*-expressing neurons. Given that *Nr5a2* is also expressed in *Agrp-negative* neuronal subtypes, it will be important to investigate its transcriptional targets in other neuronal populations in the ARC. In addition, *Nr5a2* has recently been implicated as playing an important role in the maintenance of neuronal differentiation and identity in the hippocampus. In these studies, deletion of *Nr5a2* in the dentate gyrus cells in vivo leads to a reduction of the number of NeuN and calbindin-positive neurons [89]. Similar studies in the hypothalamus will be necessary to reveal if there is a broader function of *Nr5a2* in mammalian brain function and plasticity.

To enable transcription factors to bind, chromatin must be in an “open state”. These accessible regions can be determined using Assay for Transposase-Accessible Chromatin combined with sequencing (ATAC-seq). ATAC seq studies from the human prefrontal cortex found enriched motifs for NR5A2 target genes in schizophrenia patients (treated with APs) compared with matched case controls [90]. These studies confirm that APs impact NR5A2 function in the human brain and suggest NR5A2 is an important target for future therapeutic development.

In summary, these studies identify a novel mechanism underlying OLZ-induced hyperphagia. We show that OLZ triggers the transcription of *Agrp* through NR5A2 in a subset of *Agrp-*expressing neurons to promote overconsumption and weight gain. These findings can be used to inform future clinical development of APs that do not activate hyperphagia and provide deep insights into the regulation of eating behavior. Importantly, it is critical to mitigate AP-induced weight gain to prevent patient non-compliance [91] and avoid further exacerbating the growing obesity epidemic and the associated increase in the prevalence of metabolic diseases.

### Supplementary information

supplemental figures and legends
