# Supplementary material for: Nuclear receptor 5A2 regulation of Agrp underlies olanzapine-induced hyperphagia
Source: Mol Psychiatry. 2023 Feb 10;28(5):1857–67. doi: 10.1038/s41380-023-01981-9 (PMC10412731; doi:10.1038/s41380-023-01981-9)
Supplement: Supplementary file 1 — supplemental figures and legends [file 41380_2023_1981_MOESM1_ESM.pdf]

Supplemental figure 1.

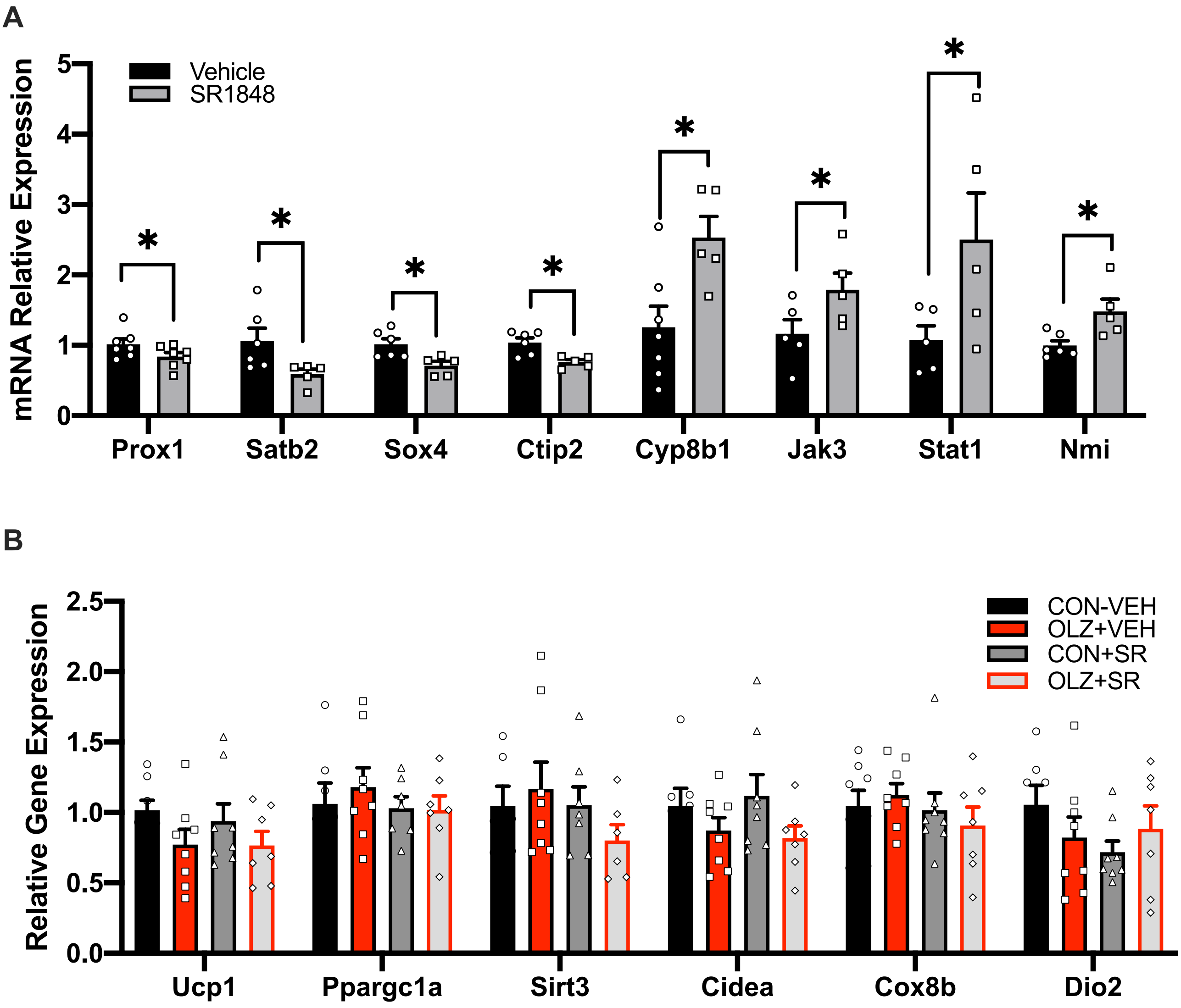

supplemental figure 2

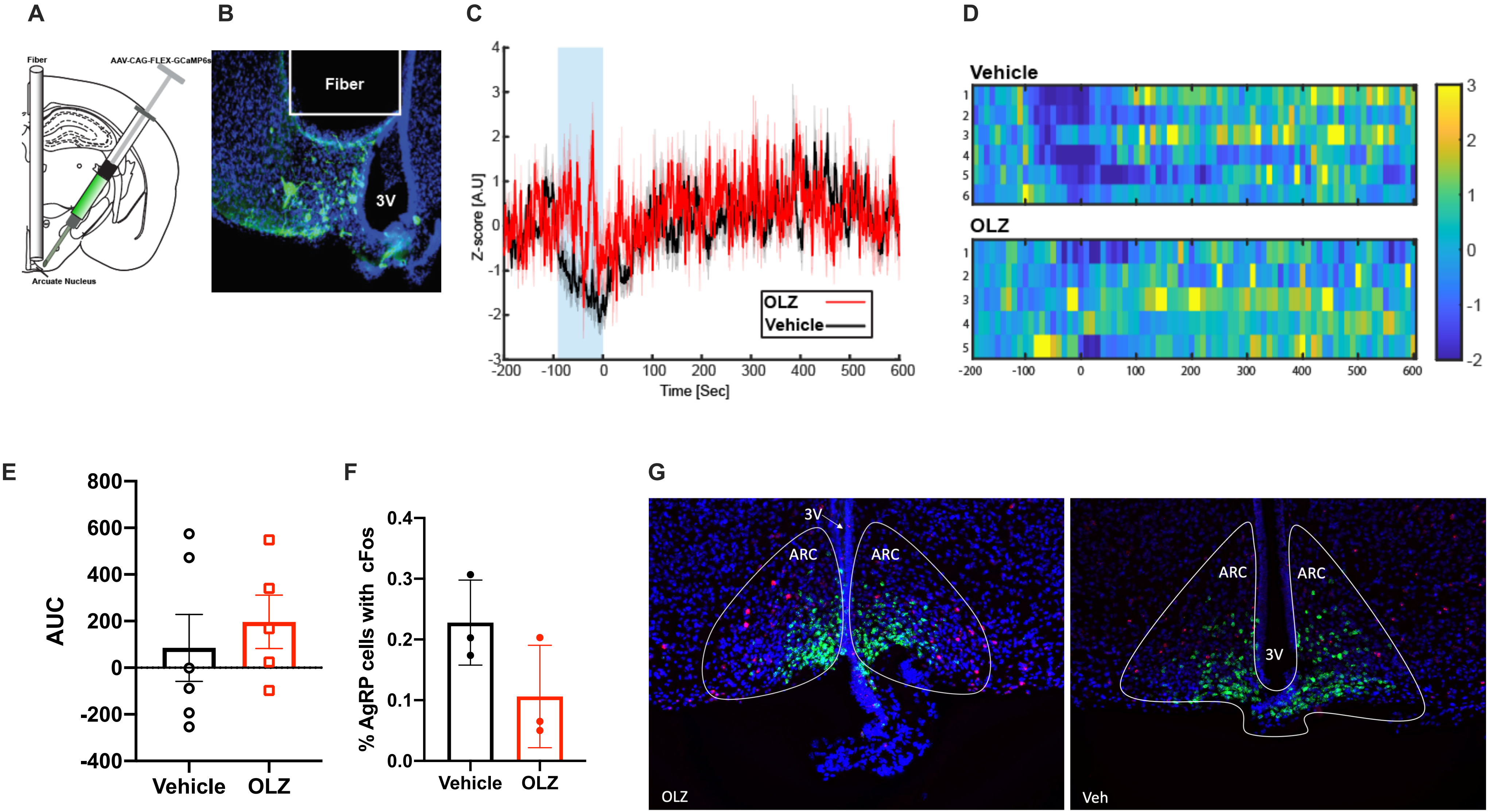

Supplemental figure 1. Systemic *Nr5a2* antagonist treatment impact on gene expression. **A.** Hypothalamic expression of NR5A2 target genes after SR1848 (30mg/kg) or vehicle treatment for 7 days, n=6-7 mice per group). Data is expressed as mean ± SEM and was analyzed using students t-test, * denotes statistical significance at $p < 0.05$. **B.** Thermogenic brown adipose tissue gene expression in WT mice fed either control diet (CON) or OLZ diet and injected with NR5A2 antagonist (SR1848, 30mg/kg) or vehicle (VEH) for 7 days. Data passed the Shapiro-Wilk test for normality and is expressed as mean ± SEM and was analyzed using either one-way ANOVA followed by Two-stage linear step-up procedure of Benjamini, Krieger and Yekutieli with a false discovery rate of 0.10. * denotes statistical significance at $p < 0.05$, n= 7-8 replicates per group.

Supplemental Figure 2 – Acute OLZ injection does not impact AGRP neuron activity. **A.** Schema of viral infusion and fiber placement. **B.** Histological verification of fiber placement and viral expression. Blue - DAPI staining, Green - AgRP neurons expressing GCaMP6s, 3V - third ventricle. **C.** Effect of acute injection of olanzapine (red, n=5) or vehicle (black, n=6) on AGRP neuron activity. Blue shaded area represents time of handling. Time zero represents mouse introduced back to recordings chamber following injection. Data presented as average +/- SEM. **D.** Heatmaps depicting individual mouse photometry dynamics, with each bin representing the average Z-score over 10 sec. Each row represents an individual mouse. Heatmap range was capped between 3 to -2 z-score values. **E.** Area under the curve for of both saline and olanzapine injected mice, following end of injection, unpaired t-test, p=0.57, (average +/- SEM), OLZ, n=5, VEH, n=6. **F.** Percent of colocalization Agrp+/Fos+ in the ARC of vehicle and OLZ-treated mice. **G.** Representative images for the RNA fluorescent in situ hybridization (FISH).
